# Paclitaxel Magnetic Core–Shell Nanoparticles Based on Poly(lactic acid) Semitelechelic Novel Block Copolymers for Combined Hyperthermia and Chemotherapy Treatment of Cancer

**DOI:** 10.3390/pharmaceutics11050213

**Published:** 2019-05-03

**Authors:** Evi Christodoulou, Maria Nerantzaki, Stavroula Nanaki, Panagiotis Barmpalexis, Kleoniki Giannousi, Catherine Dendrinou-Samara, Makis Angelakeris, Eleni Gounari, Antonis D. Anastasiou, Dimitrios N. Bikiaris

**Affiliations:** 1 Laboratory of Polymer Chemistry and Technology, Department of Chemistry, Aristotle University of Thessaloniki, 54124 Thessaloniki, Greece; evicius@gmail.com (E.C.); marinera002@msn.com (M.N.); sgnanaki@chem.auth.gr (S.N.); 2 Department of Pharmaceutical Technology, School of Pharmacy, Aristotle University of Thessaloniki, 54124 Thessaloniki, Greece; pbarmp@pharm.auth.gr; 3 Laboratory of Inorganic Chemistry, Department of Chemistry, Aristotle University of Thessaloniki, 54124 Thessaloniki, Greece; klegia@chem.auth.gr (K.G.); samkat@chem.auth.gr (C.D.-S.); 4 Department of Physics, Aristotle University of Thessaloniki, 54124 Thessaloniki, Greece; agelaker@auth.gr; 5 Biohellenika Biotechnology Company, Leoforos Georgikis Scholis 65, 57001 Thessaloniki, Greece; eleni-790@hotmail.com; 6 School of Chemical and Process Engineering, University of Leeds, Leeds LS2 9JT, UK; A.Anastasiou@leeds.ac.uk

**Keywords:** poly(lactic acid), polyesters, block copolymers, nanocarriers, magnetic core–shell nanoparticles, chemotherapy, Paclitaxel, hyperthermia, drug delivery

## Abstract

Magnetic hybrid inorganic/organic nanocarriers are promising alternatives for targeted cancer treatment. The present study evaluates the preparation of manganese ferrite magnetic nanoparticles (MnFe_2_O_4_ MNPs) encapsulated within Paclitaxel (PTX) loaded thioether-containing ω-hydroxyacid-co-poly(d,l-lactic acid) (TEHA-co-PDLLA) polymeric nanoparticles, for the combined hyperthermia and chemotherapy treatment of cancer. Initially, TEHA-co-PDLLA semitelechelic block copolymers were synthesized and characterized by ^1^H-NMR, FTIR, DSC, and XRD. FTIR analysis showed the formation of an ester bond between the two compounds, while DSC and XRD analysis showed that the prepared copolymers were amorphous. MnFe_2_O_4_ MNPs of relatively small crystallite size (12 nm) and moderate saturation magnetization (64 emu·g^−1^) were solvothermally synthesized in the sole presence of octadecylamine (ODA). PTX was amorphously dispersed within the polymeric matrix using emulsification/solvent evaporation method. Scanning electron microscopy along with energy-dispersive X-ray spectroscopy and transmission electron microscopy showed that the MnFe_2_O_4_ nanoparticles were effectively encapsulated within the drug-loaded polymeric nanoparticles. Dynamic light scattering measurements showed that the prepared nanoparticles had an average particle size of less than 160 nm with satisfactory yield and encapsulation efficiency. Diphasic PTX in vitro release over 18 days was observed while PTX dissolution rate was mainly controlled by the TEHA content. Finally, hyperthermia measurements and cytotoxicity studies were performed to evaluate the magnetic response, as well as the anticancer activity and the biocompatibility of the prepared nanocarriers.

## 1. Introduction

The smart design and tailoring properties of several nanoparticle based drug delivery systems (DDS) has led to their extensive investigation as an alternative new approach for targeted and more effective treatment of cancer [1,2]. These nano-based systems may be prepared from either organic (such as biocompatible polymers), or inorganic (such as iron oxides) materials, although recent attempts are focused on the development of novel systems combining both [3,4,5]. Generally, nanoparticles suited for targeted cancer therapy have a particle size between 1 and 200 nanometers and, depending on the materials used and the manufacturing process employed, they may possess unique physicochemical and mechanical properties [6,7,8,9]. These properties may be tailored to meet specific requirements by altering several attributes such as nanoparticle composition, size, shape, surface morphology, etc. Some of the best known advantages of nano-based carriers are the successful delivery of hydrophobic active pharmaceutical ingredients (APIs) in high doses, the protection API instability within the body, the targeted delivery of APIs for single cell or tissue therapy, the reduction in API induced systemic toxicity, the simultaneous delivery of APIs and diagnostic agents for combination therapies, etc. [10,11,12,13].

Until now, several attempts have been made for passive and active targeting of anticancer drugs, including self-triggered drug release as a result of a signal specific at the site of treatment (such as presence of specific enzymes or pH changes at the target site) or by externally activated drug release from the carrier (such as the application of light, temperature, magnetic field, and ultrasound) [14,15,16]. Among them, nanoparticle devices designed for hyperthermia treatment seem to gain increased attention in recent years [17,18,19]. Specifically, hyperthermia induced cancer therapy refers to a small temperature rise (from 41 to 45 °C) which leads to cell death through the initiation of a series of pro-apoptotic and apoptotic signaling cascades [20]. 

Magnetic particles, which have been used in hyperthermia treatment of cancer since 1979 [21], are capable of transforming electromagnetic energy from an alternating magnetic field (AMF) to heat. Recently, magnetic hyperthermia has achieved a great success in clinics, as it has been approved in Europe for the treatment of primary or recurrent glioblastoma multiform, a lethal brain tumor with limited treatment options [3]. One of the most important advantages of magnetic hyperthermia treatment is the ability of the AMF to penetrate into deeper tissues than other heat-generating sources (i.e., light or acoustic waves) [2]. In addition, it has been recently proved that hyperthermia makes also the targeted cancer cells more sensitive to alternative treatments such as chemotherapy [22]. Hence, the combination of hyperthermia and chemotherapy through the use of magnetic core–shell coated with drug loaded nanoparticles is becoming a highly effective clinical reality for cancer therapy [23,24,25,26]. In addition, such sophisticated drug delivery systems may be successfully used in ‘theranostics’, a combination approach of therapeutic and diagnostic functions within a single system, which uses diagnosis to aid or guide nanoparticle therapy [2]. In such systems, biocompatible coating of magnetic nanoparticles, besides being the carrier for the selected anticancer drug, also provides a barrier that protects the metal core from biodegradation, improves its biocompatibility, and restricts its agglomeration [23,27,28,29].

Among the several inorganic and organic materials used in the preparation of such nanoparticle systems, magnetic iron oxides in combination with biocompatible polymers such as poly(caprolactone) (PCL), poly(lactic-co-glycolic acid) (PLGA), and poly(lactic acid) (PLA) seem to gain the most attention as they show high biocompatibility, low toxicity, good capability for cell targeting, imaging and therapeutics, including attributes that activate the immune response and inhibit tumor growth [2,25,30,31,32]. In addition, both materials show an increased capability of surface modification via several agents, which help to overcome and avoid the reticuloendothelial system [33,34,35]. However, all these polyesters have their low in vivo hydrolyzability as a disadvantage. 

The aim of the present study is to prepare magnetic core–shell drug-loaded nanoparticles coated with new biocompatible polymers based on PLA for the combined hyperthermia and chemotherapy treatment of cancer. Specifically, this is the first time that the combination of MnFe_2_O_4_ magnetic nanoparticles with novel synthesized semitelechelic block copolymers of thioether-containing ω-hydroxyacid (TEHA) and poly(d,l-lactic acid) (TEHA-co-PDLLA) carrying Paclitaxel (PTX, used as a model anticancer drug) was studied. PTX is an anticancer drug used to treat a number of types of cancer [36].

## 2. Materials and Methods 

### 2.1. Materials and Reagents

10-undecenoic acid (98%), 2-mercaptoethanol (99%), and 2-dimethoxy-2-phenylacetophenone (DMPA) (99%) used during TEHA UV-irradiation preparation were purchased from Sigma-Aldrich Co. (St. Louis, MO, USA). Tin(II) 2-ethylhexanoate (Sn(Oct)_2_) (95%) purchased from Sigma-Aldrich Co. (St. Louis, MO, USA) and d,l-lactide (3,6-Dimethyl-1,4-dioxan-2,5-dion), (99%, Alfa Aesar, Karlsruhe, Germany) were used for the synthesis of the biocompatible polymers. All reagents were used without any further purification. Iron (III) acetylacetonate (Fe(acac)_3_), octadecylamine (ODA, purity >90.0%), purchased by Fluka (Seelze, Germany) and manganese (II) acetylacetonate (Mn(acac)_2_, ≥99.9%) used for the synthesis of MnFe_2_O_4_ MNPs were purchased from Sigma-Aldrich Co. (St. Louis, MO, USA. Paclitaxel (PTX), a white, odorless, crystalline powder with 99.5% purity, melting point of 213 °C and 853.91 g/mol molecular weight, used as a model drug, was purchased from Sigma-Aldrich Co. (St. Louis, MO, USA). Poly(vinyl alcohol) (PVA) with M_w_ = 9000–10,000 (80% hydrolyzed) and sodium cholate hydrate (BioXtra, ≥99%) used as stabilizers during the preparation of polymeric nanoparticles were purchased from Sigma-Aldrich Co. (St. Louis, MO, USA). DMEM high glucose/l-glutamine/sodium pyruvate, phosphate-buffered saline (PBS), Penicillin-Streptomycin Solution 100X, fetal bovine serum (FBS), and Trypsin-EDTA 0.05% in PBS were purchased from Biosera (Nuaille, France), while the (3-(4,5-Dimethylthiazol-2-yl)- 2,5-diphenyltetrazolium bromide) powder—MTT and dimethyl sulfoxide (DMSO) were purchased also from Sigma-Aldrich Co. (St. Louis, MO, USA). All other materials and solvents used in the analytical methods were of analytical grade.

### 2.2. Synthesis and Characterization of PDLLA and TEHA-co-PDLLA Copolymer

#### 2.2.1. Synthesis of Biocompatible Polymers

TEHA was synthesized based on a previously published UV-irradiation method [37,38]. In brief, 10-undecenoicacid (15 g, 81 mmol) and 2-mercaptoethanol (6.36 g, 81 mmol) was irradiated in dichloromethane solution at λ = 365 nm in the presence of DMPA (2% TEHA/initiator molar ratio) as photo-initiator. The completion of the reaction was confirmed after 60 min by the monitoring, through ^1^H-NMR, the complete disappearance of C=C double bonds. 

Neat PDLLA was synthesized using ring opening polymerization. Briefly, 10 g of dry d,l-lactide have been dissolved in 10 mL of toluene and were added in a 25 mL reaction flask. The Sn(Oct)_2_ catalyst (at a 1% *w*/*w* to d,l-lactide) in toluene solution was injected into the flask and the apparatus was evacuated several times and filled with nitrogen in order to remove oxygen. The solution was stirred until homogeneity was reached and thereafter heated at 160 °C for 1 h under a slow nitrogen flow to remove toluene and to prepare the homopolymer. Then, the melt was heated at 180 °C for 15 min under high vacuum (10^−3^–10^−6^ Torr), which was applied slowly over a period of time of about 10 min to avoid excessive foaming, in order to remove unreacted d,l-lactide and left to cool at room temperature.

**Figure 1:**
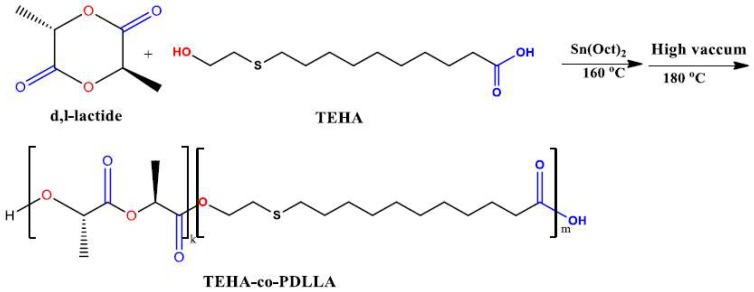
Synthesis route of TEHA-co-PDLLA semitelechelic block copolymer.

For the preparation of TEHA-co-PDLLA semitelechelic block copolymers, a mixture of dry d,l-lactide and TEHA in several molar ratios (1:5, 1:12, 1:50, 1:70, 1:100, and 1:140, TEHA to d,l-lactide) was placed in a 100 mL round bottom flask and the apparatus was evacuated several times and filled nitrogen in order to remove oxygen. Polymerization was carried out using Sn(Oct)_2_ as a catalyst (1 wt % of Sn(Oct)_2_ to d,l-lactide) under nitrogen atmosphere and constant stirring at 160 °C for 1 h. The reaction was continued up to 180 °C under high vacuum (10^−3^–10^−6^ Torr), which was applied slowly over 10 min to avoid excessive foaming, in order to remove unreacted d,l-lactide. Polymerization (Figure 1) was stopped after cooling to room temperature.

#### 2.2.2. Characterization of Biocompatible Block Polymers

Nuclear magnetic resonance (^1^H-NMR) spectra of the copolymers were obtained using a Bruker spectrometer (Billerica, MA, USA) operating at a frequency of 400 MHz for protons. The samples were dissolved in deuterated chloroform (CDCl_3_) at 5% w/v and spectra were recorded at 20 °C with tetramethylsilane (TMS) as an internal standard. The number of scans was 10 and the sweep width was 6 kHz.

Fourier transform infrared (FTIR) spectra were obtained using a Perkin–Elmer FTIR spectrometer (model Spectrum One, Perkin Elmer, Dresden, Germany). The materials were in the form of thin films with thickness of ~30 μm and the spectra were collected in the region of 400–4000 cm^−1^ using a resolution of 4 cm^−1^ and 64 co-added scans.

Wide angle X-ray scattering (WAXD) study, in the form of thin films, was performed over the range 2θ from 5° to 60°, at steps of 0.05° and counting time of 5 s, using a MiniFlex II X-ray diffractometry (XRD) system from Rigaku Co. (Chalgrove, Oxford, UK) with CuKa radiation (*l* = 0.154 nm).

Differential scanning calorimeter (DSC) study performed using Pyris Diamond DSC (Perkin–Elmer, Dresden, Germany), calibrated with indium and Zinc standards. Samples of 5.0 ± 0.1 mg sealed in aluminum pans were heated up to 180 °C at a heating rate of 20 °C/min under nitrogen atmosphere and held at that temperature for 1 min. Then the samples were cooled to 30 °C at a cooling rate of 300 °C/min, held at this temperature for 2 min and subsequently heated again up to 180 °C with 20 °C/min in order to record the glass transition (*T*_g_) and melting point (*T*_m_) temperatures. 

Size exclusion chromatography (SEC, PL-GPC 220; Agilent Technologies, Santa Clara, CA, USA) equipped with an isocratic pump Spectra System P1000 (Thermo Fischer, Waltham, MA, USA), column oven Model 605 (SSI LabAlliance, State College, PA, USA), three columns in series (PLgel10μm, MIXED-B, 300 × 7.5 mm) purchased by Agilent (Santa Clara, CA, USA), a refractive index detector (Shodex RI-101; Showa Denko America Inc., New York, NY, USA), and ultraviolet absorbance detector (SpectraSYSTEM UV1000; Thermo Fischer, Waltham, MA, USA) was used for the determination of molecular weight. Orthodichlorobenzene (oDCB) was used as eluent while the system was calibrated with eight PS standards (Mp: 4500–3,400,000 g mol^−1^). In every case, prior to calculating the polydispersity indices (PDI) of the unknown materials as well as prior to making an estimation on the average molecular weights (Mn and Mw), a series of standard PS solutions were tested in order to examine the accuracy of the measurements.

### 2.3. Synthesis and Characterization of MnFe_2_O_4_ MNPs

#### 2.3.1. Synthesis of MnFe_2_O_4_ MNPs

MnFe_2_O_4_ MNPs were prepared in an autoclave by the decomposition of acetylacetonate iron (III) and manganese (II) in a 2:1 ratio, Fe(acac)_3_ 1.8 mmol:Mn(acac)_2_ 0.9 mmol in the presence solely of ODA 12.9 mmol. The temperature of the oven was elevated at a steady rate 4 °C/min and was raised to 200 °C and kept stable for 24 h. After the 24 h reaction, the autoclave was left to cool down to room temperature with a rate of 5 °C/min and MnFe_2_O_4_ MNPs were isolated after repeated washing cycles with EtOH and centrifugation (5000 rpm).

#### 2.3.2. Characterization of MnFe_2_O_4_ MNPs

Powder X-ray diffraction (XRD) was performed using a Rigaku (Chalgrove, Oxford, UK) Ultima diffractometer (40 kV, 30 mA, CuKa radiation) with Bragg–Brentano geometry (detection limit 2% approximately). Fourier transform infrared spectroscopy (280–4000 cm^−1^) was recorded using a Nicolet FTIR 6700 spectrometer (Thermo Scientific, Waltham, MA, USA), with samples prepared as KBr pellets. Thermogravimetric analysis (TGA) was performed using a SETA-RAM SetSys1200 (SETARAM Instrumentation, Caluire, France) instrument at a heating rate of 10 °C/min under N_2_ atmosphere. Magnetic measurements were acquired by a vibrating sample magnetometer (1.2H/CF/HT; Oxford Instruments Ltd. (Abingdon, Oxfordshire, UK).

### 2.4. Preparation of Polymeric Nanoparticles and Core–Shell Magnetic Nanoparticles

Polymer nanoparticles with (or without) Paclitaxel were prepared by the emulsification solvent evaporation method. Initially, neat polymeric nanoparticles were prepared by dissolving 50 mg of the TEHA-co-PDLLA block copolymer in 2 mL of dichloromethane (DCM) and homogenized using a probe sonicator model UP50H (Hielscher Ultrasound Technology, Teltow, Germany) at 15 W for 2 min with an aqueous phase containing either a) 10 mL of 0.5% w/v PVA solution, or b) 6 mL of 12 mM sodium cholate hydrate solution. The O/W emulsion formed was gently stirred at room temperature under a fume hood until the evaporation of the organic solvent was completed. Nanoparticles were purified by centrifugation at 9500 rpm for 20 min and reconstituted from the precipitate in fresh water (twice). The resulting suspension was lyophilized (Scanvac, Coolsafe 110-4 Pro, Labogen, Scandinavia) and stored at ambient temperature under vacuum until further study. Nanoparticles with addition of PTX were also prepared based on the above process by adding proper amounts (25 mg in 250 mg of polymer) of PTX in the organic DCM solution. Additionally, for the preparation of core–shell magnetic nanoparticles, 10 mg of superparamagnetic manganese ferrite nanoparticles were added in the PTX-polymer DCM solution and the process was following as described above.

### 2.5. Characterization of Nanoparticles and Core–Shell Magnetic Nanoparticles

The surface morphology of the polymeric nanoparticles and the magnetic nanoparticles encapsulated within the polymer nano-matrix was determined by scanning electron microscopy SEM/Energy-dispersive X-ray spectroscopy (SEM/EDS) using the FEI Helios NanoLab 650 (Thermo Fischer Scientific, Waltham, MA, USA) operated at 5 kV. Crystallographic data on the iron oxide nanoparticles were obtained by X-ray powder diffraction analysis using a Guinier Camera G670 (Huber, Rimsting, Germany) (CuKa1 radiation transmission geometry, Ge (1 1 1) monochromator on a primary beam, Ge external standard) in the 2θ-range 10–100°. 

Bright-field scanning transmission electron microscopy (BF-STEM) investigations were performed on carbon coated samples using a Hitachi SU8230 microscope (Chiyoda, Tokyo, Japan), equipped with a cold field emission gun (1–30 kV).

FTIR spectra and WAXD diffractograms were obtained using the same organology and conditions as described above.

Particle size distribution of the prepared nanoparticles was determined by dynamic light scattering (DLS) using a Zetasizer Nano instrument (ZEN 3600; Malvern Instruments, Malvern, Worcestershire, UK) operating with a 532 nm laser. A suitable amount of nanoparticles was dispersed in distilled water creating a total concentration of 1‰ and kept at 37 °C before the measurement. For each sample, five measurements have been done.

Differential scanning calorimeter (DSC) study performed using a Perkin–Elmer, Pyris Diamond DSC (Dresden, Germany), calibrated with indium and zinc standards. Samples of 5.0 ± 0.1 mg sealed in aluminum pans were heated up to 190 °C at a heating rate of 20 °C/min under nitrogen atmosphere and held at that temperature for 1 min in order to erase any thermal history. Then, the samples were cooled to 0 °C at a cooling rate of 200 °C/min in order to prevent re-crystallization and then re-heated again up to 230 °C with the same heating rate (20 °C/min). From this second DSC scan, the glass transition temperatures (T_g_) or melting point (T_m_) of the samples was recorded.

For magnetic hyperthermia measurements the commercially available 4.5 KW ultrahigh frequency induction heating machine of Shuangping (Model SPG-06AB-III, Shuangping, China) was used. Time-temperature measurements were recorded with a step of 0.4 s, during heating (300 s) under a 25 mT (250 Oe) alternating magnetic field, at 765 kHz. Also, sample’s cooling curves were recorded for another 300 s. Three ferrite suspensions (MnFe_2_O_4_ MNPs) at 5, 10, and 20 mg/mL of water were measured in total, while 1 mL of the initial suspension was initially homogenized in an ultrasonic bath for 5 min.

### 2.6. Drug Release Studies

High performance liquid chromatography (HPLC) was utilized for drug content determination. In particular, a Shimadzu HPLC system (model LC-20AD, Tokyo, Japan) was used consisting of a degasser (Model DGU-20A5, Tokyo, Japan), a pump (Model LC-20AD, Tokyo, Japan), a manual injector with a 100 μL loop (Model Rheodyne, Cotati, CA, USA), a variable wavelength UV–vis detector (Model SPD-20A, Tokyo, Japan), and a column oven (Model CTO-20AC, Tokyo, Japan). 3 mg of nanoparticles were added in 50 mL of ACN/H_2_O 50/50 v/v and stirred until complete solubilization. A clear solution was obtained which was filtered through 45 μm filters prior to HPLC analysis. A ZORBAX Eclipse XDB-C18 (Agilent, Santa Clara, CA, USA) 5 μm, 250 × 4.6 mm analytical column was used, while the flow rate was set at 1 mL/min and the column temperature was maintained at 25 °C. A photodiode array detector (model SPD-M20A, Shimadzu, Tokyo, Japan) was used at 227 nm and the quantification of PTX was based on a calibration curve prepared at 20, 10, 5, 2.5, 1, and 0.5 μg/mL PTX to mobile phase (water/ACN 30/70 v/v). The nanoparticle yield, g and drug entrapment efficiency (EE) were calculated using the equations

Yield (%) = [weight of nanoparticles]/[initial weight of polymers and PTX] × 100(1)

EE (%) = [weight of PTX in nanoparticles]/[initial weight of PTX] × 100(2)

In vitro drug release studies were performed using the Distek Dissolution Apparatus (Evolution 2100C, North Brunswick Township, NJ, USA). Drug-loaded nanoparticle suspensions corresponding to 2 mg of drug were placed in a dialysis cellulose membrane bag having a molecular weight cut-off of 12,400, tied and placed into the baskets. Dissolution medium consisted of 500 mL simulated body fluid (pH = 7.4) having ion concentration essentially equal to those of human blood plasma (as described by Kokubo and Takadama [1,39]) with the addition of Tween 80 (0.1% *w*/*w*) and the stirring rate was kept constant at 50 rpm, as was the temperature at 37 ± 0.5 °C. At predetermined time intervals, 2 mL of the aqueous solution was withdrawn from the release media. The samples were filtered and assayed for drug by the HPLC method described above. In each experiment, the samples were analyzed in triplicate.

### 2.7. Cytotoxicity Studies

#### 2.7.1. Caco-2 and hASCs Cell Culture

To perform all cytotoxicity tests, Biohellenika SA (Thessaloniki, Greece) provided Caco-2 colorectal cancer cell line, while human mesenchymal stem cells derived from adipose tissue (adipose derived stem cells-ASCs) isolated as described below. Briefly, during lipectomy adipose tissue was collected in PBS (phosphate buffered saline) (pH 7.4) after the consent of a healthy volunteer donor. After washing with saline and gentle lice cutting, overnight lysis was performed with 50 μg collagenase in a moving incubator. The next day the mixture was filtered via a 70 μm pores’ filter and centrifuged at 850 g for 10 min at room temperature. The pellet was resuspended in Dulbecco’s modified Eagle’s medium (DMEM) supplemented with 10% Fetal Bovine Serum (FBS) and 2% penicillin/streptomycin and plated in culture flasks for 72 hours until cells’ adherence to the plastic surface (37 °C incubation with 5% CO_2_). Both Caco-2 and ASCs were incubated at 37 °C in a humidified incubator with 5% CO_2_ and cultured for several passages. Medium was changed every two days. Cells were then detached with 0.05% Trypsin-EDTA and counted with a Neubauer chamber for plating in 24-well plates.

#### 2.7.2. Sterilization of Nanoparticles

After weighing the nanoparticles, two control concentrations: 200 and 1000 μg/mL (corresponding to approximately 2 to 10 uM of PTX) in DMEM medium supplemented with 10% FBS and 2% penicillin/streptomycin were prepared to be added directly into the cell culture. Filters of 0.22 μm pore size were used to sterilize the solutions.

#### 2.7.3. Measuring Cytotoxicity Levels with the MTT Test after 24 Hours Incubation

To determine the cytotoxicity effect of both nanoparticles, the MTT assay was performed 24 h after the initial coating of the cells in 24-well plates. Day 0 was the day of the addition of nanoparticles in culture supernatants for both cell lines. Briefly, after supernatant removal from the wells, MTT reagent was added at a ratio 1:10 in DMEM medium followed by 4 h incubation (37 °C, 5% CO_2_). The MTT was removed and 1 mL/well of dimethyl sulfoxide (DMSO) was added for one more hour for incubation under the same conditions. Absorbance was measured at 570 nm and 630 nm (UV–vis spectrophotometer; Perkin Elmer, Dresden, Germany).

## 3. Results and Discussion

### 3.1. Characterization of Biocompatible Polymers

**Figure 2:**
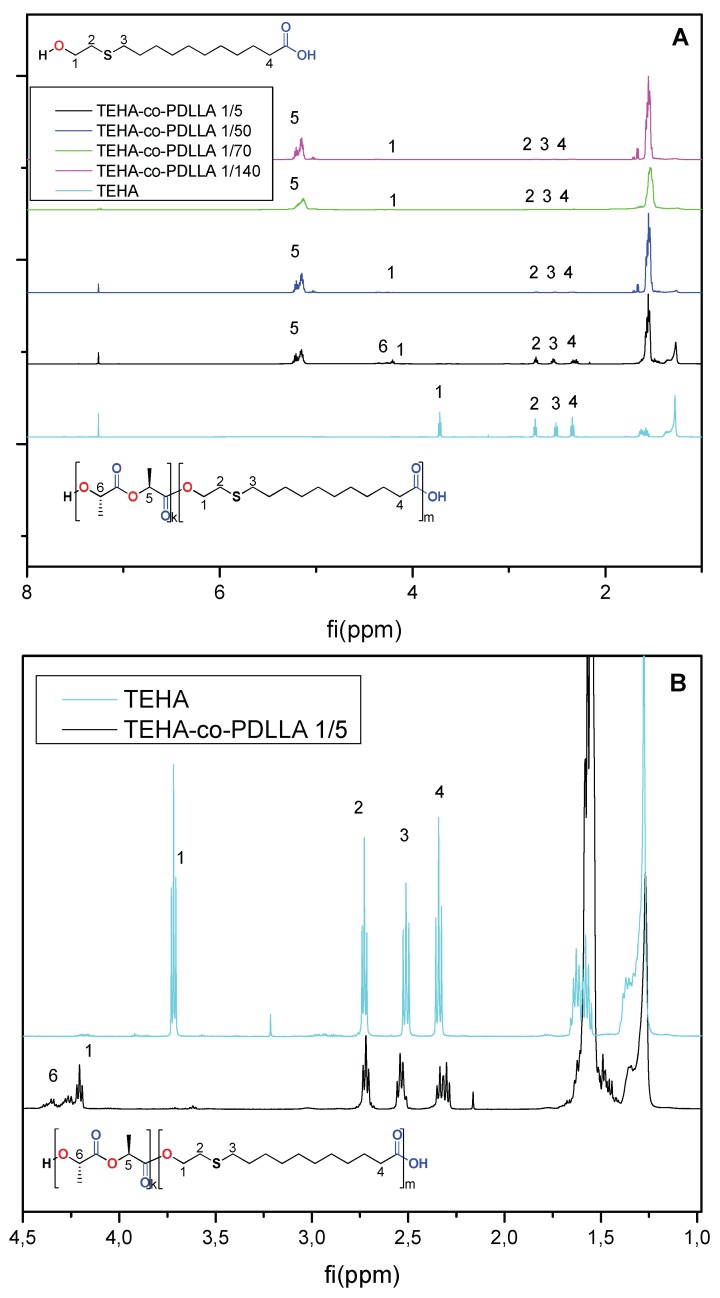
^1^H NMR spectra of (**A**) neat TEHA and TEHA-co-PDLLA semitelechelic block copolymers and (**B**) TEHA and TEHA-co-PDLLA 1/5 copolymer with higher magnification.

Figure 2 shows the ^1^H NMR spectra of the neat TEHA and the prepared TEHA-co-PDLLA semitelechelic block copolymers. Regarding the neat TEHA, peaks recorded at 3.72 (1), 2.73 (2), 2.51 (3), and 2.34 (4) ppm correspond to the hydrogen protons of (t, 2H, –CH_2_–OH), (t, 2H, –CH_2_–S–), (m, 2H, –CH_2_–S–), and (t, 2H, –CH_2_–CO), respectively, while peaks at 5.15 (5) and 4.32 (6) ppm in the spectrum of TEHA-co-PDLLA, correspond to the hydrogen protons of (t, 1H, –O–CH–CO) and (t, 1H, HO–CH–CO) of PDLLA. Additionally, a shift in the TEHA hydrogen protons corresponding to the –CH_2_–OH group from 3.72 to 4.18 ppm indicated the formation of an ester bond (–CH–CO–O–CH_2_) between TEHA and PDLLA. This is an evidence that copolymers between PDLLA and TEHA have been formed. Since TEHA has only one hydroxyl groups, this can act as initiator for ring opening polymerization of lactide monomer and, according to Figure 1, only block copolymers can be formed since the –COOH end group of TEHA is inactive during ROP of lactide. Similar block copolymers have been reported in literature using methylated poly(ethylene glycol) as initiator for ring opening polymerization reactions [40,41]. The formation of a copolymer is supported also by two additional observations. In the region between 4.1 and 4.5 ppm, three peaks can be distinguished. One of them corresponds to the –CO–***CH***(CH_3_)–OH end group of TEHA-co-PDLLA. The other two are from the methylene group of TEHA adjacent to carbonyl groups, the most important corresponds to the CH_2_ group between two adjacent TEHA units, while the smallest to the CH_2_ group of a TEHA unit adjacent to a PDLLA unit. This is a clear indication that ester groups between TEHA and PDLLA were formed. 

The calculated number-based average molecular weights of copolymers are in agreement with the initial feed ratios and were found to be 12,200, 29,000, 45,800 and 86,200 for 1/5, 1/50, 1/70 and 1/140 TEHA to PDLLA ratios, respectively. As can be seen, by increasing TEHA content the Mn is reduced since TEHA (due to its hydroxyl groups) acts as initiator for ring opening polymerization of cyclic oligomers like d,l-lactide [38,42]. The greater the number of these groups the lower the molecular weight of produced copolymers, and hence, the TEHA-co-PDLLA 1/5 copolymer with the highest TEHA content has the lowest molecular weight compared to the rest copolymers. PDI of all copolymers is too high, but in ring opening polymerizations is very common to produce copolymers with such values, especially for low reaction times as in our case [43].

**Figure 3:**
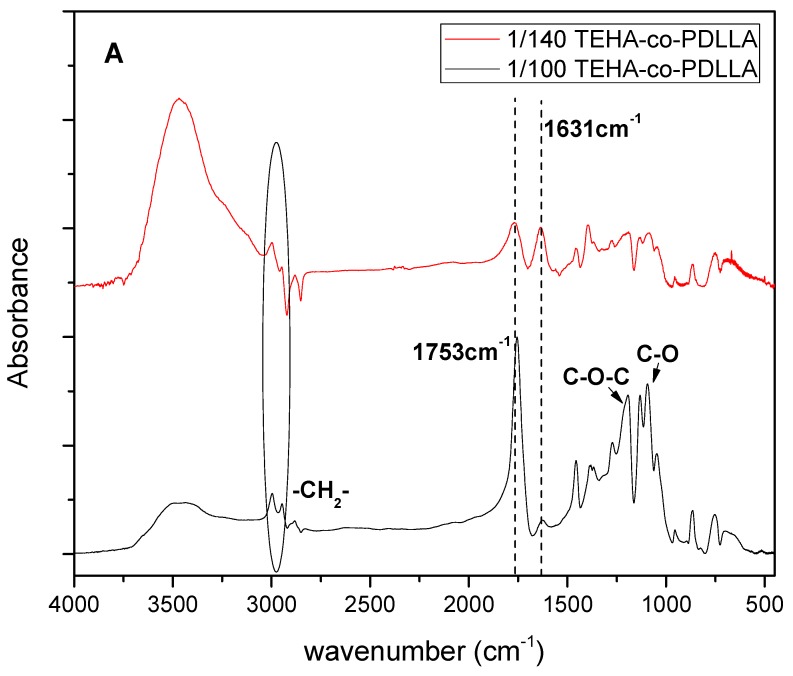

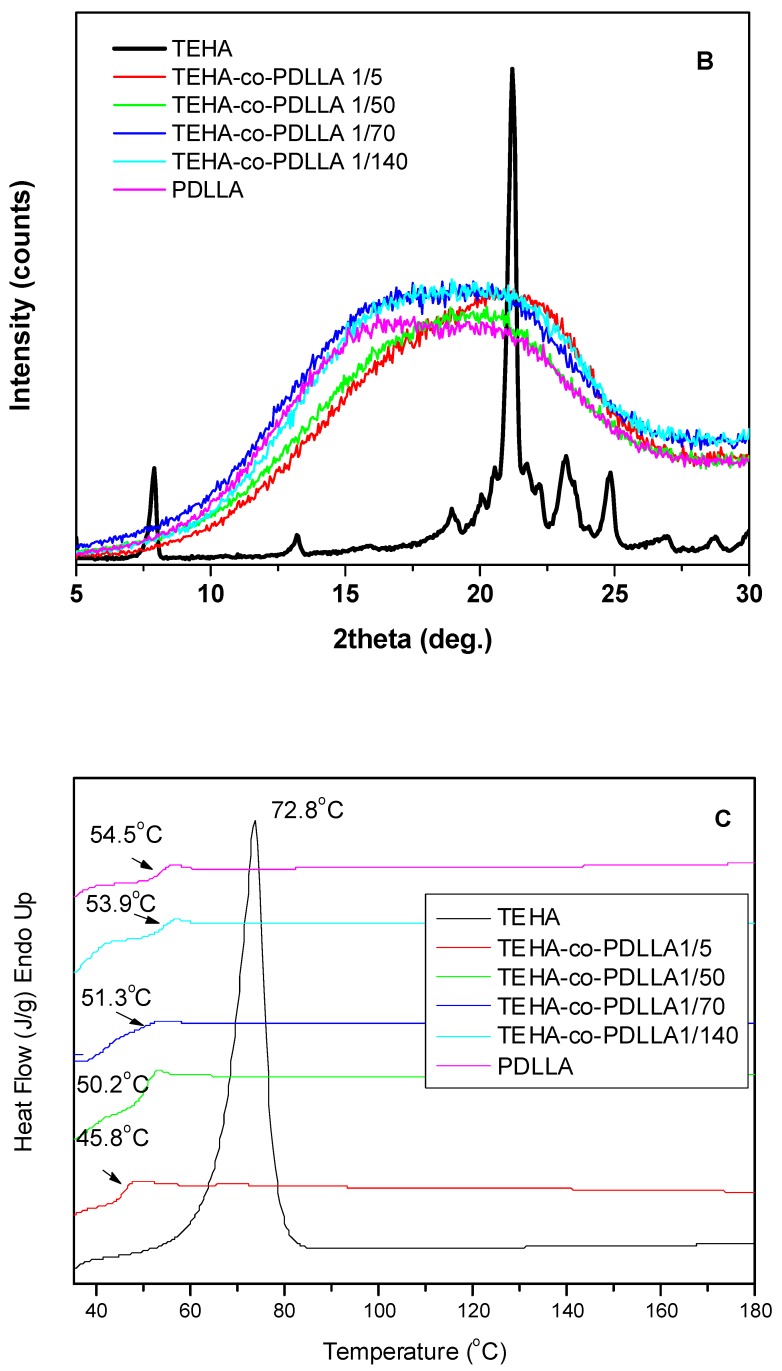
Neat TEHA, neat PDLLA, and TEHA-co-PDLLA semitelechelic block copolymers characterized via FTIR (**A**), XRD (**B**), and DSC second heat scan (**C**) analysis.

The formation of copolymers was also verified from FTIR spectra of prepared polymers (Figure 3A). Results indicate that the intensity of peaks corresponding to –CH_2_ (~2900 cm^−1^) and –C=O (1753 cm^−1^) groups of TEHA increase as the content of TEHA increases. Additionally, in the case of high TEHA to PDLLA ratio the peaks located in the region of 1195 to 1090 cm^−1^, corresponding to the C–O–C and C–O of TEHA and PDLLA ester groups, are shown more sharply due to the formation of the ester bond (–CH–CO–O–CH_2_) between the two compounds during copolymerization. All these copolymers, as was found from XRD studies, are completely amorphous.

Figure 3B shows the XRD diffractograms of the prepared copolymers in comparison with those of neat TEHA and PDLLA. TEHA is a high crystalline compound with peaks at 2θ 21.2°, 23.1°, and 24.8°, while the synthesized PDLLA is amorphous. Additionally, all prepared TEHA-co-PDLLA block copolymers were amorphous, since an amorphous halo was observed in all prepared samples (verified also by DSC analysis, see below in the same paragraph). This absence of crystallinity was expected since PDLLA is well known that crystalizes very difficult [44] and only if the crystallization time is long enough [45]. This amorphization is desired and expected to enhance PDLLA’s slow degradation rate, which is a main drawback in PDLLA based nano (or micro) particle formulations. The amorphous structure of copolymers is also evident in DSC thermograms. DSC first heating scan (data not shown) revealed that the neat TEHA shows an endothermic melting peak, while pure PDLLA as well as the prepared TEHA-co-PDLLA copolymers were completely amorphous since no melting peaks were recorded. Additionally, due to its slow crystallization rate, there is also no cold crystallization recorded, which can be seen in other polylactide polymers with other stereoregularity like PLLA [46]. Furthermore, Figure 3C shows the DSC thermograms of the second heating scan, where it is clear that increasing TEHA concentration in copolymers led to materials with progressively decreasing T_g_ values, indicating that the ω-hydroxyacid is acting as a plasticizer to the PDLLA. Thus, the copolymer with the highest TEHA amount (TEHA-co-PDLLA 1/5) has the lowest T_g_ value (45.8 °C).

### 3.2. Characterization of MnFe_2_O_4_ MNPs

**Figure 4:**
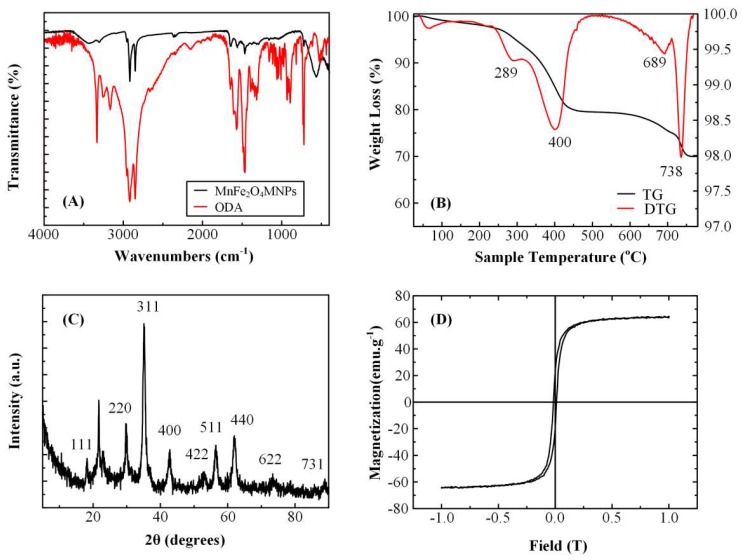
FTIR spectra (**A**), TG and DTG curves (**B**), XRD pattern (**C**), and VSM measurements (**D**) of MnFe_2_O_4_ MNPs.

The presence of the organic coating on the MNPs surface was certified by FT-IR spectroscopy and thermogravimetric analysis (Figure 4A,B, respectively). The FT-IR spectra of MnFe_2_O_4_ MNPs and neat ODA are given in Figure 4A. The N–H stretching and wagging modes at ~3303 cm^−1^ and ~725 cm^−1^ respectively observed in the FTIR spectrum of MnFe_2_O_4_ MNPs are assigned to ODA, while they are slightly downshifted compared to the same features of neat ODA (~3336 cm^−1^, 715 cm^−1^), indicating the attachment of ODA on the metal core. The asymmetric and symmetric stretching vibrations of the methylene groups are observed at ~2954, 2841, and 2931 cm^−1^, respectively, while peaks at ~1460 and ~1650 cm^−1^ are assigned to bending modes of –CH_2_ and –NH_2_ groups. Finally, the peaks at ~560 and 380 cm^−1^ are characteristic of Fe-O and Mn-O vibrations respectively, in the spinel structure [42].

Thermogravimetric data analysis in Figure 4B showed that the weight loss (~30%) was performed in two main steps suggesting the existence of a bilayer structure surrounding the NPs and/or different binding sites of the functional groups of ODA. The hydrocarbon chain decomposition occurred at 200–450 °C, while the removal of the amine group took place at higher temperatures owing to the bonding with the metal core. 

Powder X-ray diffraction diagrams of MnFe_2_O_4_ MNPs showed all characteristic peaks of the Jacobsite syn space group Fd-3m (227) MnFe_2_O_4_ (Figure 4C). The peak at 21° is attributed to the crystallization of the surfactant, while the absence of any other peaks highlights the pure manganese ferrite phase. The average crystallite size of the sample was calculated by fitting the diffraction data with a pseudo-Voigt function (Jade6 Software) and was found 12 nm. Figure 4D depicts the magnetic features of MnFe_2_O_4_ MNPs by VSM measurements at a maximum field of 1 T. The Ms and the coercive field (Hc) value were 45 emu·g^−1^ and 260 Oe, respectively. By taking into account the percentage of ODA found by TGA analysis, the corrected Ms value was found 64 emu·g^−1^ per metallic core [47,48].

### 3.3. Characterization of Polymeric Nanoparticles

**Figure A1:**
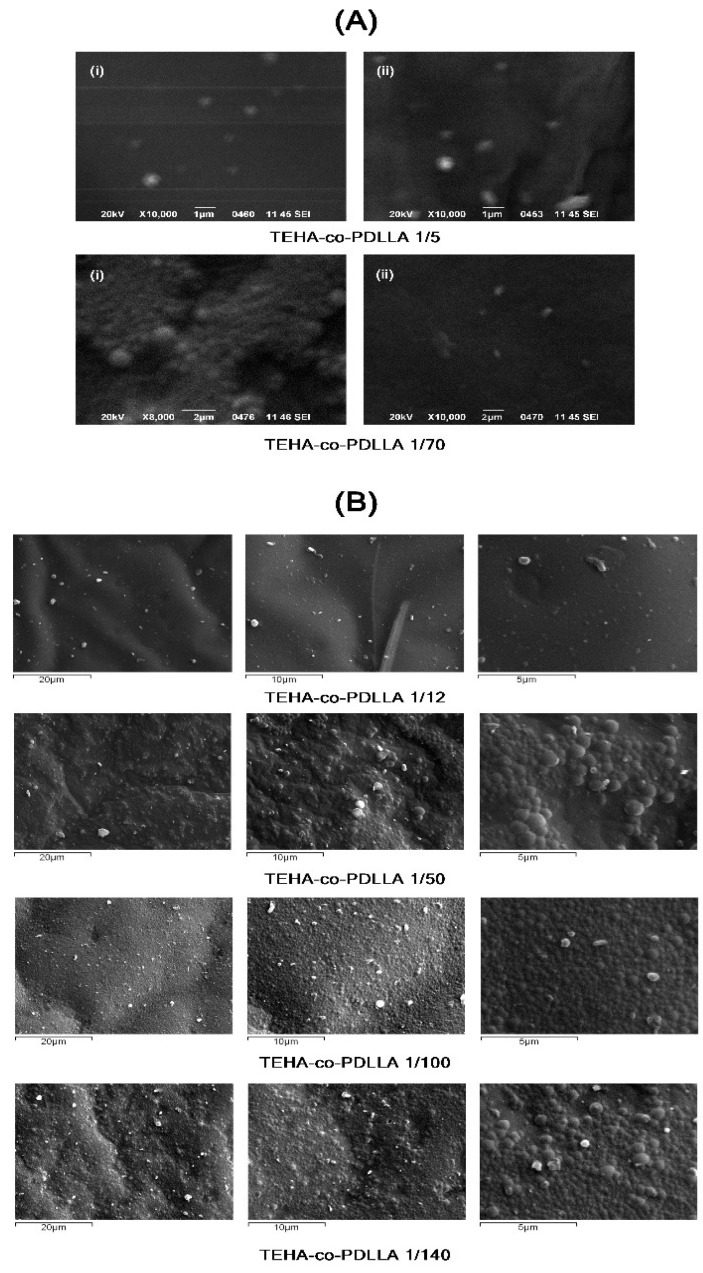
SEM micrographs of (**A**) prepared neat nanoparticles having PVA 0.5 w/v (i) or sodium cholate hydrate 12 mM (ii) in the aqueous phase and (**B**) prepared neat nanoparticles having PVA 0.5 w/v in the aqueous phase at varying TEHA to PDLLA ratios.

Preliminary experiments were conducted in order to identify the optimum polymeric nanoparticle formulation parameters in regards to emulsifier type and molar ratio of TEHA to PDLLA. For this reason, all prepared nanoparticles have been studied and characterized by several techniques. SEM micrographs of the prepared neat nanoparticle (without PTX) having PVA 0.5 w/v or sodium cholate hydrate 12 mM as emulsifiers are shown in Appendix A (Figure A1A(i,ii)). Results show that in the case of sodium cholate hydrate increased irregularities and agglomeration of nanoparticles was obtained, indicating the sodium cholate hydrate is not able to adequately stabilize the prepared emulsion. For this reason, PVA was chosen as stabilizer and Figure A1B shows also the SEM micrographs of the neat nanoparticles prepared at varying TEHA to PDLLA ratio, where improvement in terms of shape and yield (i.e., number of nanoparticles per micrograph) were observed with increasing PDLLA content. From DLS measurements it was found that TEHA-co-PDLLA 1/100 copolymers give nanoparticles with 113 ± 12 nm average size diameter, while the copolymer with 1/140 ratios gives nanoparticles with 125 ± 15 nm average size diameter. This increase may be attributed to the hydrophobic properties of PDLLA and the hydrophilic effect of TEHA which due to its surface carboxyl groups is expected to act as an emulsifier. Additionally, increasing PDLLA content led to the formation of higher molecular weight copolymers (Table 1), which may in turn lead to nanoparticles with higher particle sizes. 

**Figure 5:**
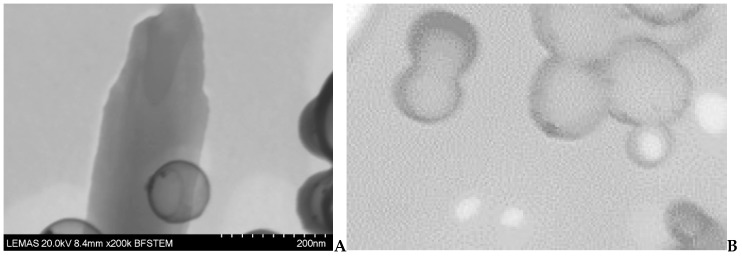
TEM micrographs of TEHA-co-PDLLA 1/140 copolymers in (**A**) low and (**B**) higher magnification.

In general, the prepared TEHA-co-PDLLA copolymers, are block copolymers consisted from a hydrophobic PDLLA part and a hydrophilic TEHA part. These blocks will behave differently during the emulsification solvent evaporation procedure used for the preparation of nanoparticles. It is expected that the hydrophobic PDLLA part will be dissolved and placed inside of formed DCM droplets while the hydrophilic TEHA, due to its –COOH groups, will be at the surface of these droplets and close to the water phase, acting as an emulsifier. When DCM evaporates and nanoparticles harden (taking their final shape) this disposition will lead to the formation of core–shell type nanoparticles, with the hydrophobic PDLLA located in the core and the hydrophilic TEHA forming the shell. To prove this hypothesis TEM micrographs were taken (Figure 5) were the core–shell type of nanoparticle was verified by the thin (due to TEHA’s MW~149 g/mol) but darker surface layer surrounding the lighter (but much larger in size) core (PDLLA) of the prepared nanoparticles. Similar core–shell nanoparticles are expected when the superparamagnetic manganese ferrite nanoparticles are added into the system (discussed in following parts of the manuscript). 

**Figure 6:**
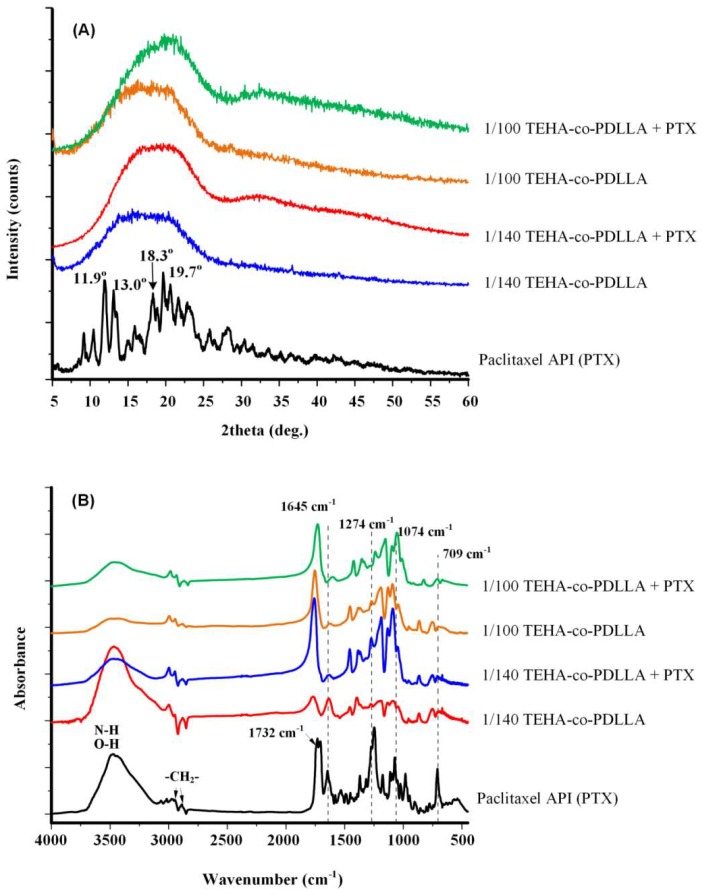
XRD difractograms (**A**) and FTIR spectra (**B**) of Paclitaxel and TEHA-co-PDLLA nanoparticles.

In a further step, the physical state of the prepared nanoparticles was evaluated. For this reason, XRD analysis was used to characterize the physical state of PTX within the polymeric matrix. As it can be seen from pattern in Figure 6A the neat PTX is a high crystalline compound (most characteristic XRD peaks are recorded at 11.9°, 13.0°, 18.3°, and 19.7°). All prepared nanoparticles showed only an amorphous halo indicating that PTX is probably dispersed in amorphous phase. Furthermore, the shape of recorded curves is similar to that of the neat copolymers. This amorphization could be the result of interactions between polymeric macromolecules and PTX, since the drug has a lot of carbonyl, hydroxyl, and secondary amine groups that could interact with the hydroxyl end groups of PDLLA or with its ester groups, respectively. In order to identify if there are any interactions occurring between PTX and the copolymers, FTIR spectroscopy (Figure 6B) was used. In the case of PXT, characteristic IR peaks are recorded in 3494–3300, 2976–2888, 1732, 1645, 1248, and 1274 cm^−1^ corresponding to the >NH and –OH, >CH_2_, >C=O, –C–O–C–, and –C–N– groups of PTX, while in the case of the drug-loaded nanoparticles the recorded 1274 cm^−1^ peak, corresponding to the –C–N– group of PTX, indicates the presence of PTX within the system. Examining the spectra of drug encapsulated nanoparticles, it can be seen that these peaks are recorded at the same wavenumber as in the case of neat drug, while there are no shifts recorded in the characteristic peak absorbencies of copolymers. Hence, from it is clear that no specific interactions are taking place between the drug and the copolymers. 

**Figure A2:**
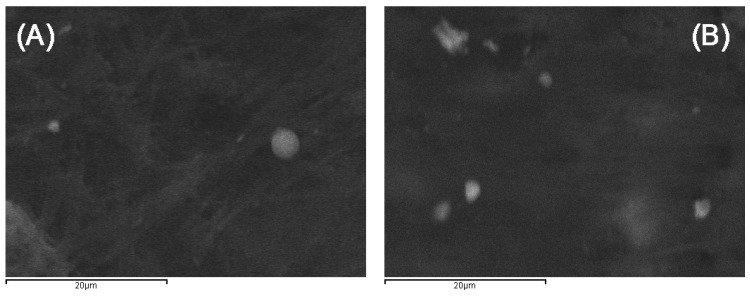
SEM micrograms of Paclitaxel loaded TEHA-co-PDLLA nanoparticles for 1/70 (**A**) and 1/140; (**B**) TEHA to PDLLA.

Figure A2 (Appendix A) shows the SEM micrographs of the drug-loaded nanoparticles, where, in both TEHA to PDLLA ratios tested, nanoparticles were formed with a size ranging from 150 to 300 nm. Additionally, in the case of 1/100 ratio the formed nanoparticles had a less irregular shape compared to 1/140 ratio, while the nanoparticles with the latter ratio showed increased yield (i.e., number of nanoparticles per micrograph).

### 3.4. Characterization of Magnetic Core–Shell Drug-Loaded Nanoparticles

**Figure 7:**
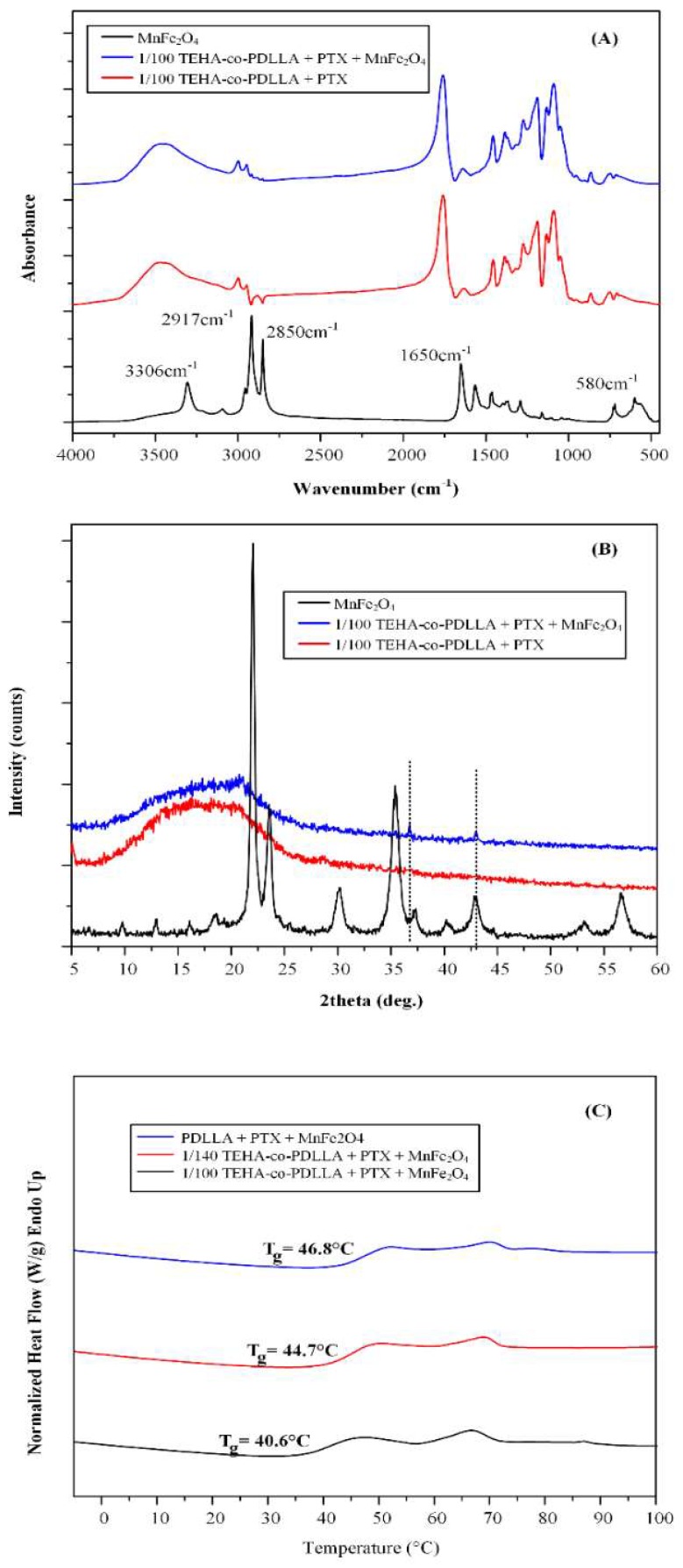
FTIR spectra (**A**), XRD diffractograms (**B**), and DSC thermograms (**C**) of magnetic core–shell drug-loaded polymeric nanoparticles.

Initially, FTIR analysis (Figure 7A) was used in order to identify the presence of encapsulated MnFe_2_O_4_ MNPs within the polymeric matrix in accordance with the initial MnFe_2_O_4_ NPs for comparison reasons. The magnetic core–shell polymeric nanoparticles did not show any characteristic peaks of MnFe_2_O_4_ MNPs probably due to the low content of the latter. Hence, in order to verify the presence of MnFe_2_O_4_ MNPs within the system, XRD analysis was used (Figure 7B). Results showed mainly an amorphous halo located at ~10° to 25° attributed to the polymer, while two MnFe_2_O_4_ characteristic peaks of low intensity, indicates that the prepared magnetic nanoparticles were effectively encapsulated within the polymeric matrix. Additionally, DSC analysis of the samples in Figure 7C showed that the T_g_ of the prepared polymers (recorded at 40.6, 44.7, and 46.8 °C for 1/100 TEHA-co-PDLLA, 1/140 TEHA-co-PDLLA, and neat PDLLA, respectively) increases proportionally to the content of PDLLA. This is in good agreement with the already observed T_g_ shift, recorded for nanoparticles without the encapsulation of magnetite nanoparticles.

**Figure 8:**
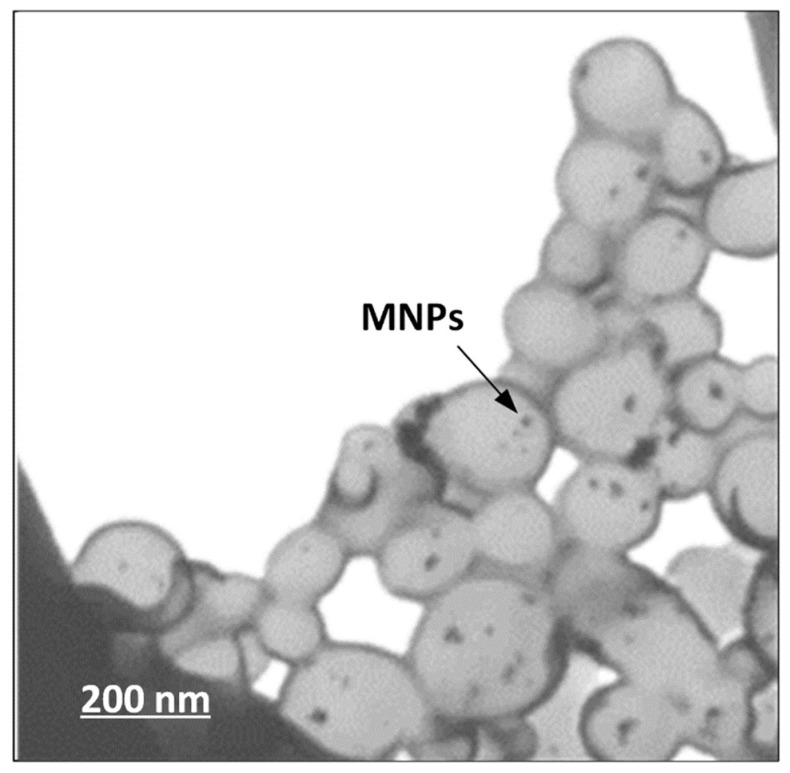
BF-STEM images of the drug-loaded polymeric MnFe_2_O_4_ nanoparticles.

**Figure A3:**
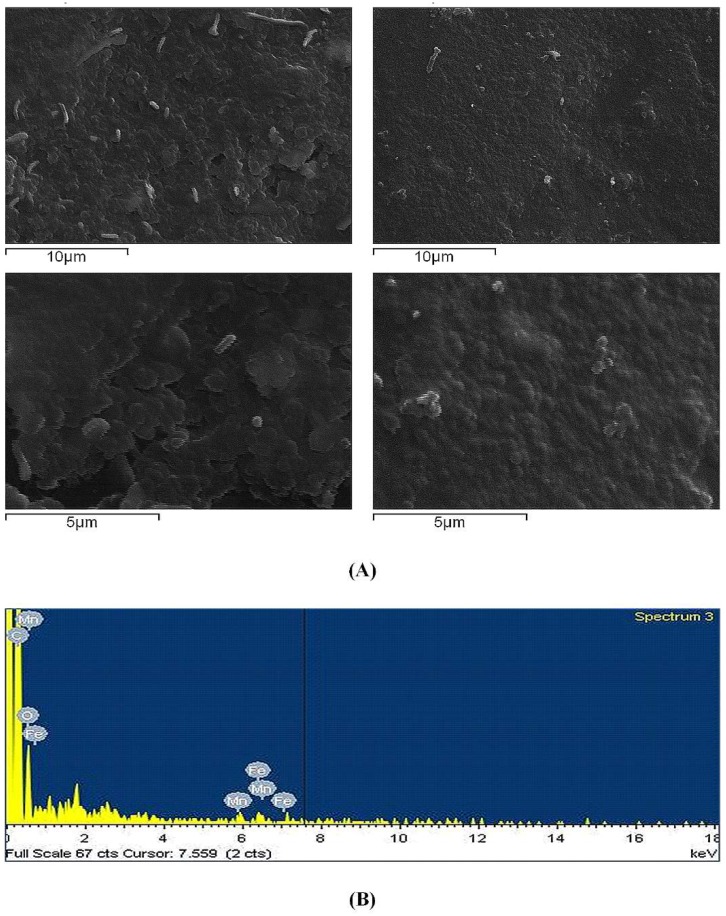
SEM and EDS analysis of the prepared magnetic core–shell drug-loaded polymeric nanoparticles.

In order to verify that the magnetic nanoparticles were effectively encapsulated within the drug-loaded polymeric nanoparticles, SEM/EDS and BF-STEM analysis were used. Figure A3 (Appendix A) shows the SEM/EDS images for the prepared systems. Generally, SEM analysis provides information about the shape, the size of the particles investigated while it can reflect accurately the topography of the surface, revealing the three-dimensional structure. In addition, when combined with EDS, SEM/EDS analysis may provide an excellent technique for identification of specific compounds within the studied system. In the case of the magnetic core–shell drug-loaded polymeric nanoparticles, Figure A3A showed changes in the shape and size compared to the neat drug-loaded polymeric nanoparticles. Specifically, the incorporation of the magnetic nanoparticles led to irregularly shaped nanoparticles with increased size and harsh surface. Additionally, elemental analysis performed on the prepared systems (Figure A3B) showed the presence of Mn, O, and Fe, elements which are found in the chemical structure of the magnetic nanoparticles. Therefore, SEM analysis demonstrated the prepared particles were in the nanoscale, while the use of EDS verified the presence of MnFe_2_O_4_ MNPs within the examined system. However, as it is not clear whether the MnFe_2_O_4_ nanoparticles are encapsulated within the polymeric nanoparticles or located in their surface, BF-STEM imaging was used (Figure 8). In contrast to the initial polymeric nanoparticles (Figure 5) where homogeneous particles were prepared with small irregularities in shape, in the case of MnFe_2_O_4_ loaded polymeric nanoparticles an increase in size was observed with more irregularities in shape. Additionally, some degree of agglomeration between the prepared nanoparticles is observed, probably due to attractive forces induced by the presence of MnF_e2_O_4_ NPs within the system. Finally, from Figure 8 it is verified that the MnFe_2_O_4_ nanoparticles were actually encapsulated within the polymeric nanoparticles and can be seen as black spots inside nanoparticles. Additionally, the same figure verifies that the core–shell type of the prepared TEHA-co-PDLLA nanoparticles, observed in Figure 5, was not affected by the addition of both PTX and the MnFe_2_O_4_ MNPs.

The mean particle size of the magnetic core–shell polymeric nanoparticles, as well as their distribution, was measured by dynamic light scattering. Particle size of the prepared nanoparticles is one of the most important parameters regarding drug release, physical stability, and cellular uptake [23,49]. Especially in hyperthermia cancer treatment, it is desired to develop nanoparticles with a mean particle size above 100 nm, in order to take advantage of the physical changes occurring in the tumor tissue. All prepared nanoparticles showed unimodal size distribution (data not shown), with mean particle sizing of 111, 124, and 152 nm, for TEHA-co-PDLLA 1/100, TEHA-co-PDLLA 1/140, and neat PDLLA, respectively. Hence, results showed that all prepared systems may work effectively with respect to their size as hyperthermia agents. Additionally, due to the hydrophilic nature and the ability of TEHA, an increase in TEHA content leads to a decrease in mean particle size of nanoparticles. Zeta potential is also slightly decreased, compared with neat PDLLA, however, all values are very close. It is well known that ζ-potential is depending from many parameters included pH and ionic strength, polymer hydrophobicity, etc. For similar copolymers like PLGA it was reported a ζ-potential about −54.2 mV and when a hydrophilic polymer like PEG was used to prepare block copolymers, the ζ-potential was shifted from −2 to −7 mV [50,51]. In a further step, nanoparticle yield content and % EE were evaluated (Table 2).

Yield varied from 58.6% to 75.6% indicating high process efficacy when the content of PDLLA increases, this may be attributed to the hydrophobic nature of the polymer and the plasticizing effects of TEHA, while EE varied from 5.28% (in the case of neat PDLLA) to 6.23% (in the case of TEHA-co-PDLLA 1/100). Generally, several factors may affect % EE, such as the affinity of the loaded drug with the polymer system, the hydrophobicity of the polymer matrix, drug solubility in water, drug–drug interaction (i.e., its ability to self-aggregate), etc. Specifically, in the present study, E.E. increased with increasing TEHA content, probably due the latter’s hydrophilic nature which aids in acting as an emulsifier/stabilizer during the preparation of polymeric nanoparticles.

**Figure 9:**
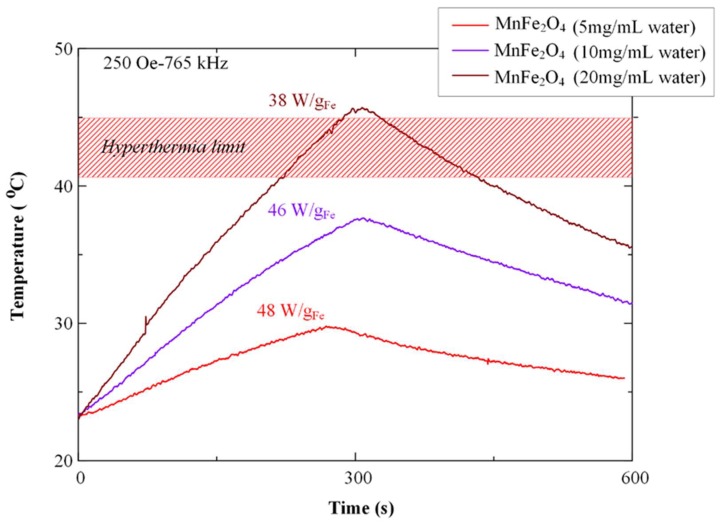
Hyperthermia curves for the prepared magnetic core–shell nanoparticles.

Figure 9 shows the hyperthermia effect (temperature vs. time) of the prepared magnetic core–shell polymeric nanoparticles in the exposure of a magnetic field.

Generally, three types of hyperthermia treatment are being identified, namely thermo ablation, moderate hyperthermia, and diathermia [52]. In the first case, application of high temperatures in the range of 46 °C < T < 56 °C lead to tumor cells necrosis, however normal tissues are also damaged during treatment. In moderate hyperthermia (which is the most preferable for cancer treatment), the application of temperatures between 41 and 45 °C is selected [53]. Finally, diathermia finds application in the field of physiotherapy by applying temperatures lower than 41 °C [54]. As it is depicted in Figure 9, the MnFe_2_O_4_ MNPs respond to the magnetic field and a clear temperature rise was measured. It was also identified that a concentration around 20 mg MnFe_2_O_4_ MNPs/mL H_2_O is required in order to reach the temperature for moderate hyperthermia treatment (41–45 °C). This indicates their potential of combination therapy in cancer treatment, but further studies should be carried out to determine the optimum concentration of MNPs in the final formulation.

**Figure 10:**
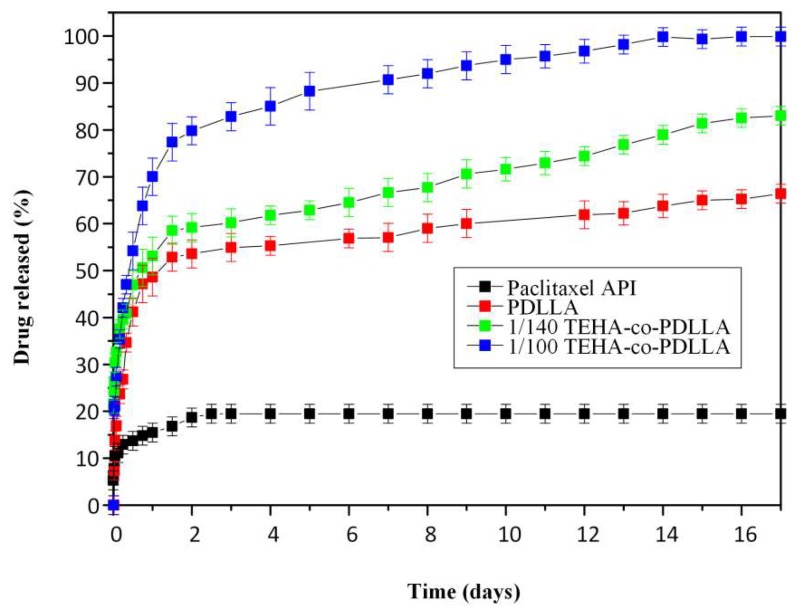
PXT dissolution profiles for the prepared magnetic core–shell polymeric nanoparticles.

Dissolution studies in Figure 10 showed that the pure PTX exhibits a very low dissolution rate and extent (not exceeding 20% PTX release even after 18 days of testing) which can be attributed to the low aqueous solubility and the hydrophobic nature of PXT. In contrary to pure PTX, all nanoparticle formulations showed much higher dissolution release rate and extent, which can be attributed to the amorphous dispersion of PTX inside the polymer matrix. In all cases, PTX release from the prepared systems followed a two-step release kinetics, with an initial burst release observed up to approximately 48 h (due to the surface bonded PTX) followed by a controlled release from 48 h till the end of dissolution process (in 18 days). Additionally, results showed that neat PDLLA nanoparticles exhibit lower dissolution rate and extent compared to nanoparticles containing TEHA. Specifically, in the case of PDLLA nanoparticles, 66.2% of the PTX was released after 18 days of dissolution compared to TEHA-co-PDLLA 1/140 and 1/100 where 82.8% and 99.9% of PTX was released in the same time point. Generally, such differences may be attributed to several factors, such as water permeability and solubility within the polymer matrix, degree of crystallinity and crystalline morphology of polymers and PTX, hydrophilic/hydrophobic balance of the system, particle size and size distribution of the prepared nanoparticles, drug loading levels, etc. Specifically, in the present study, since PTX and copolymers are amorphous in all cases, these differences are due to the addition of TEHA. Especially, as the TEHA content increases, the hydrophilicity of the prepared matrix increases which in turn leads to increase matrix erosion rates and hence dissolution rate. Additionally, as TEHA works also as an emulsifier during the preparation of the polymeric nanoparticles, increasing concentrations of TEHA lead to smaller sized nanoparticles and hence (through the Noyes and Whitney theory [55]) to an increase of PTX dissolution rate.

**Figure 11:**
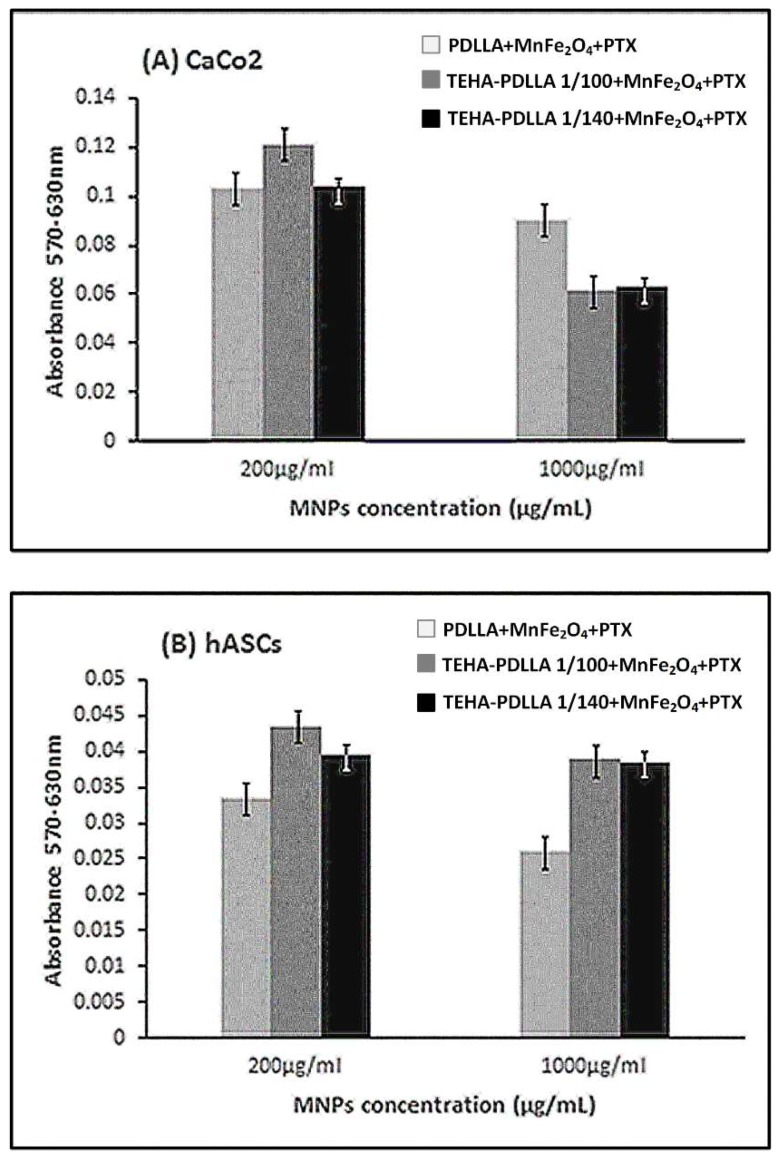
The viability of Caco-2 cells (**A**) and hASCs (**B**) incubated with MNPs. Cells viability was determined by MTT assay cells’ treatment with MNPs (200 and 1000 μg/mL) for 24 h. The absorbance values are directly proportional to metabolic activity of the cells.

**Figure 12:**
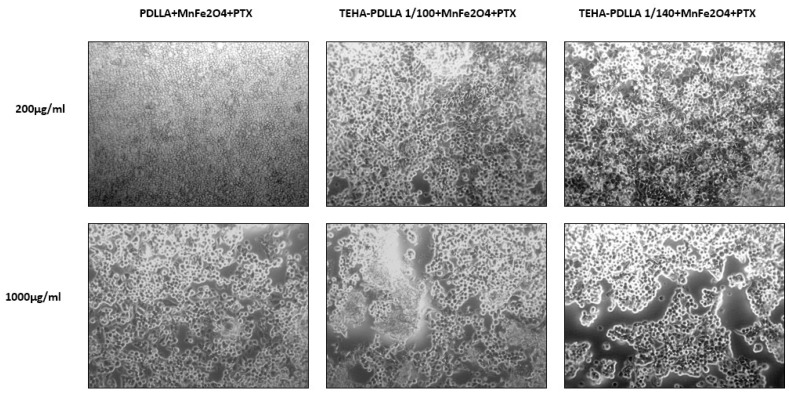
Microscope images of the cellular activity of Caco2 cells, treated with MNPs (200 and 1000 μg/mL) for 24 h.

Figure 11 demonstrates the cytotoxicity effect of the prepared magnetic nanoparticles on Caco-2 cell line and hASCs. The mitochondrial functionality upon treatment with the prepared nanoparticles is presented in Figure 11A. The Y’Y axis’ values represent the reduction of yellow 3-(4,5-dimethythiazol2-yl)-2,5-diphenyl tetrazolium bromide (MTT) by mitochondrial succinate dehydrogenase. The MTT enters the cells and passes into the mitochondria where it is reduced to formazan product. The cells are then solubilized with an organic solvent and the released, solubilized formazan reagent is measured spectrophotometrically. Since reduction of MTT can only occur in metabolically active cells the level of activity is a measure of the viability of the cells. Both TEHA-co-PDLLA based MNPs result in lower metabolic activity of the cancer cell line, compared to the neat PDLLA prepared nanoparticles. As expected, the viability was dose-dependent and was significantly decreased after treatment with higher MNP concentration (1000 μg/mL). This was also confirmed by the microscope images taken (Figure 12). By contrast, data collected from the hASCs culture (Figure 11B) showed that the absorbance values remained relatively high (especially in the case of the TEHA-co-PDLLA based nanoparticles) at both doses, a fact that indicates the rather low cytotoxicity of the prepared nanoparticles.

## 4. Conclusions

In the present study, MnFe_2_O_4_ nanoparticles were encapsulated in PXT loaded polymeric nanoparticles based on TEHA-co-PDLLA semitelechelic block copolymers. Characterization of the TEHA-co-PDLLA system showed that the prepared copolymers had an amorphous structure while TEHA acted as a plasticizer within the polymeric system (increasing TEHA content led to decreasing T_g_ values). ODA proved to have a beneficial role in the synthesis of well-defined MnFe_2_O_4_ MNPs with free amines on the surface, based on the synthetic conditions. The physicochemical characteristics of the as-synthesized MnFe_2_O_4_ MNPs such as relatively small size and moderate magnetization, allowed for further functionalization. In that vein, PXT was amorphously dispersed within the polymer matrix, while electron microscopy observations (SEM and BF-STEM) confirmed the encapsulation of the MnFe_2_O_4_ nanoparticles inside the polymeric matrix. In vitro drug release studies showed that the prepared nanoparticles were able to sustain PTX release for up to 18 days, while hyperthermia measurements confirmed the satisfactory magnetic response of MnFe_2_O_4_ MNPs and cytotoxicity studies showed that the prepared nanocarriers exhibit great anticancer activity and at the same time low toxicity toward the primary human stem cells derived from adipose tissue. Therefore, the proposed MnFe_2_O_4_ magnetic core–shell PXT loaded TEHA-co-PDLLA nanoparticles may be promising candidates in developing sophisticated drug-delivery systems for the combined hyperthermia and chemotherapy cancer treatment.

## Figures and Tables

**Table 1:** Molar masses of neat PDLLA and TEHA-co-PDLLA semitelechelic block copolymers.

Sample	Molar Ratio TEHA:d,l-lactide	Mn (g/mol)	Mw (g/mol)	PDI
PDLLA	-	147,600	470,500	3.2
TEHA-co- PDLLA 1/5	1:5	12,200	29,700	2.4
TEHA-co- PDLLA 1/50	1:50	29,000	107,000	3.7
TEHA-co- PDLLA 1/70	1:70	45,800	170,900	3.7
TEHA-co- PDLLA 1/140	1:140	86,200	231,000	2.7

**Table 2:** Particle size distribution, yield, and % EE of magnetic core–shell drug-loaded polymeric nanoparticles.

Polymer Used	Particle Size (nm)	ζ-Potential (mV)	Yield (%)	Entrapment Efficiency (%)
TEHA-co-PDLLA 1/100	111 ± 10	-32	58.6 ± 2.8	6.23
TEHA-co-PDLLA 1/140	124 ± 13	-34	75.6 ± 4.2	5.61
PDLLA	140 ± 12	-28	73 ± 1.7	5.28

## References

[1] Shi J., Kantoff P.W., Wooster R., Farokhzad O.C. (2017). Cancer nanomedicine: Progress, challenges and opportunities. Nat. Rev. Cancer.

[2] Zhang H., Liu X.L., Zhang Y.F., Gao F., Li G.L., He Y., Peng M.L., Fan H.M. (2018). Magnetic nanoparticles based cancer therapy: Current status and applications. Sci. China Life Sci..

[3] Spirou S.V., Costa Lima S.A., Bouziotis P., Vranjes-Djuric S., Efthimiadou E., Laurenzana A., Barbosa A.I., Garcia-Alonso I., Jones C., Jankovic D. (2018). Recommendations for in vitro and in vivo testing of magnetic nanoparticle hyperthermia combined with radiation therapy. Nanomaterials.

[4] Ye H., Shen Z., Yu L., Wei M., Li Y. (2018). Manipulating nanoparticle transport within blood flow through external forces: An exemplar of mechanics in nanomedicine. Proc. Soc. A: Math. Phys. Eng. Sci..

[5] Zhang W., Liu L., Chen H., Hu K., Delahunty I., Gao S., Xie J. (2018). Surface impact on nanoparticle-based magnetic resonance imaging contrast agents. Theranostics.

[6] DiStasio N., Lehoux S., Khademhosseini A., Tabrizian M. (2018). The Multifaceted Uses and Therapeutic Advantages of Nanoparticles for Atherosclerosis Research. Materials.

[7] Farina N.H., Zingiryan A., Vrolijk M.A., Perrapato S.D., Ades S., Stein G.S., Lian J.B., Landry C.C. (2018). Nanoparticle-based targeted cancer strategies for non-invasive prostate cancer intervention. J. Cell. Physiol..

[8] Gao Y., Kraft J.C., Yu D., Ho R.J.Y. (2019). Recent developments of nanotherapeutics for targeted and long-acting, combination hiv chemotherapy. Eur. J. Pharm. Biopharm..

[9] Xie Y., Wang Y., Li J., Hang Y., Oupicky D. (2018). Promise of chemokine network-targeted nanoparticles in combination nucleic acid therapies of metastatic cancer. Wiley Interdiscip. Rev. Nanomed. Nanobiotechnol..

[10] Fenton O.S., Olafson K.N., Pillai P.S., Mitchell M.J., Langer R. (2018). Advances in biomaterials for drug delivery. Adv. Mater..

[11] Ganipineni L.P., Danhier F., Preat V. (2018). Drug delivery challenges and future of chemotherapeutic nanomedicine for glioblastoma treatment. J. Control. Release.

[12] Manzano M., Vallet-Regí M. (2018). Mesoporous silica nanoparticles in nanomedicine applications. J. Mater. Sci. Mater. Med..

[13] Villegas M.R., Baeza A., Vallet-Regí M. (2018). Nanotechnological Strategies for Protein Delivery. Molecules.

[14] Bae Y.H., Park K. (2011). Targeted drug delivery to tumors: Myths, reality and possibility. J. Control. Release.

[15] Mikhail A.S., Allen C. (2009). Block copolymer micelles for delivery of cancer therapy: Transport at the whole body, tissue and cellular levels. J. Control. Release.

[16] Zensi A., Begley D., Pontikis C., Legros C., Mihoreanu L., Wagner S., Büchel C., Von Briesen H., Kreuter J. (2009). Albumin nanoparticles targeted with Apo E enter the CNS by transcytosis and are delivered to neurones. J. Control. Release.

[17] Giustini A.J., Petryk A.A., Cassim S.M., Tate J.A., Baker I., Hoopes P.J. (2010). Magnetic nanoparticle hyperthermia in cancer treatment. Nano Life.

[18] Kettering M., Grau I., Pömpner N., Stapf M., Gajda M., Teichgräber U., Hilger I. (2015). Means to increase the therapeutic efficiency of magnetic heating of tumors. Biomed. Eng./Biomed. Tech..

[19] Kobayashi T. (2011). Cancer hyperthermia using magnetic nanoparticles. Biotechnol. J..

[20] Harmon B., Takano Y., Winterford C., Gobe G. (1991). The Role of Apoptosis in the Response of Cells and Tumours to Mild Hyperthermia. Int. J. Radiat. Biol..

[21] Gordon R.T., Hines J.R., Gordon D. (1979). Intracellular hyperthermia. A biophysical approach to cancer treatment via intracellular temperature and biophysical alterations. Med. Hypotheses.

[22] Kufe D.W., Holland J.F., Frei E., Society A.C. (2003). Cancer Medicine 6.

[23] Filippousi M., Papadimitriou S.A., Bikiaris D.N., Pavlidou E., Angelakeris M., Zamboulis D., Tian H., Van Tendeloo G. (2013). Novel core–shell magnetic nanoparticles for Taxol encapsulation in biodegradable and biocompatible block copolymers: Preparation, characterization and release properties. Int. J. Pharm..

[24] Hervault A., Thanh N.T.K. (2014). Magnetic nanoparticle-based therapeutic agents for thermo-chemotherapy treatment of cancer. Nanoscale.

[25] Pradhan P., Giri J., Rieken F., Koch C., Mykhaylyk O., Döblinger M., Banerjee R., Bahadur D., Plank C. (2010). Targeted temperature sensitive magnetic liposomes for thermo-chemotherapy. J. Control. Release.

[26] Wust P., Hildebrandt B., Sreenivasa G., Rau B., Gellermann J., Riess H., Félix R., Schlag P. (2002). Hyperthermia in combined treatment of cancer. Lancet Oncol..

[27] Rong H., Xiaogang Y., Jun S., Feng G., Bifeng P., Daxiang C. (2007). Core/shell fluorescent magnetic silica-coated composite nanoparticles for bioconjugation. Nanotechnology.

[28] Salehizadeh H., Hekmatian E., Sadeghi M., Kennedy K. (2012). Synthesis and characterization of core-shell Fe_3_O_4_-gold-chitosan nanostructure. J. Nanobiotechnol..

[29] Verma N.K., Crosbie-Staunton K., Satti A., Gallagher S., Ryan K.B., Doody T., McAtamney C., MacLoughlin R., Galvin P., Burke C.S. (2013). Magnetic core-shell nanoparticles for drug delivery by nebulization. J. Nanobiotechnol..

[30] Lee J.-H., Huh Y.-M., Jun Y.-W., Seo J.-W., Jang J.-T., Song H.-T., Kim S., Cho E.-J., Yoon H.-G., Suh J.-S. (2006). Artificially engineered magnetic nanoparticles for ultra-sensitive molecular imaging. Nat. Med..

[31] Mikhaylov G., Mikac U., Magaeva A.A., Itin V.I., Naiden E.P., Psakhye I., Babes L., Reinheckel T., Peters C., Zeiser R. (2011). Ferri-liposomes as an MRI-visible drug-delivery system for targeting tumours and their microenvironment. Nat. Nanotechnol..

[32] Zanganeh S., Hutter G., Spitler R., Lenkov O., Mahmoudi M., Shaw A., Pajarinen J.S., Nejadnik H., Goodman S., Moseley M. (2016). Iron oxide nanoparticles inhibit tumour growth by inducing pro-inflammatory macrophage polarization in tumour tissues. Nat. Nanotechnol..

[33] Ma L.L., Jie P., Venkatraman S.S. (2008). Block copolymer ‘stealth’ nanoparticles for chemotherapy: Interactions with blood cells in vitro. Adv. Funct. Mater..

[34] Romberg B., Hennink W.E., Storm G. (2008). Sheddable coatings for long-circulating nanoparticles. Pharm. Res..

[35] Van Butsele K., Jérôme R., Jérôme C. (2007). Functional amphiphilic and biodegradable copolymers for intravenous vectorisation. Polymer.

[36] Rowinsky E.K., Donehower R.C. (1993). The clinical pharmacology of paclitaxel (Taxol). Semin. Oncol..

[37] Oĝuz T., Meier M.A.R. (2010). Fatty acid derived monomers and related polymers via thiol-ene (click) additions. Macromol. Rapid Commun..

[38] Nerantzaki M., Adam K.-V., Koliakou I., Skoufa E., Avgeropoulos A., Papageorgiou G.Z., Bikiaris D.N. (2017). Novel castor oil-derived block copolymers as promising candidates for biological applications: Biorelevant and biocompatible. Macromol. Chem. Phys..

[39] Kokubo T., Takadama H. (2006). How useful is SBF in predicting in vivo bone bioactivity?. Biomaterials.

[40] Nerantzaki M., Skoufa E., Adam K.-V., Nanaki S., Avgeropoulos A., Kostoglou M., Bikiaris D. (2018). Amphiphilic Block Copolymer Microspheres Derived from Castor Oil, Poly(ε-carpolactone), and Poly(ethylene glycol): Preparation, Characterization and Application in Naltrexone Drug Delivery. Materials.

[41] Panagi Z., Beletsi A., Evangelatos G., Livaniou E., Ithakissios D., Avgoustakis K. (2001). Effect of dose on the biodistribution and pharmacokinetics of PLGA and PLGA–mPEG nanoparticles. Int. J. Pharm..

[42] Georgiadou V., Makris G., Papagiannopoulou D., Vourlias G., Dendrinou-Samara C. (2016). Octadecylamine Mediated Versatile Coating of CoFe_2_O_4_ NPs for the Sustained Release of Anti-inflammatory Drug Naproxen and in vivo Target Selectivity. ACS Appl. Mater. Interfaces.

[43] Beletsi A., Leontiadis L., Klepetsanis P., Ithakissios D., Avgoustakis K. (1999). Effect of preparative variables on the properties of poly(dl-lactide-co-glycolide)–methoxypoly(ethyleneglycol) copolymers related to their application in controlled drug delivery. Int. J. Pharm..

[44] Nerantzaki M., Prokopiou L., Bikiaris D.N., Patsiaoura D., Chrissafis K., Klonos P., Kyritsis A., Pissis P. (2018). In situ prepared poly(DL-lactic acid)/silica nanocomposites: Study of molecular composition, thermal stability, glass transition and molecular dynamics. Thermochim. Acta.

[45] Xiao L., Wang B., Yang G., Gauthier M., Ghista D.N. (2012). Poly(Lactic Acid)-Based Biomaterials: Synthesis, Modification and Applications. Biomedical Science, Engineering and Technology.

[46] Papageorgiou G., Achilias D., Nanaki S., Beslikas T., Bikiaris D., Achilias D. (2010). PLA nanocomposites: Effect of filler type on non-isothermal crystallization. Thermochim. Acta.

[47] Vamvakidis K., Katsikini M., Sakellari D., Paloura E.C., Kalogirou O., Dendrinou-Samara C. (2014). Reducing the inversion degree of MnFe_2_O_4_ nanoparticles through synthesis to enhance magnetization: Evaluation of their 1 H NMR relaxation and heating efficiency. Dalton Trans..

[48] Vamvakidis K., Sakellari D., Angelakeris M., Dendrinou-Samara C. (2013). Size and compositionally controlled manganese ferrite nanoparticles with enhanced magnetization. J. Nanopart..

[49] Liggins R.T., Hunter W.L., Burt H.M. (1997). Solid-State Characterization of Paclitaxel. J. Pharm. Sci..

[50] Avgoustakis K. (2003). Effect of copolymer composition on the physicochemical characteristics, in vitro stability, and biodistribution of PLGA–mPEG nanoparticles. Int. J. Pharm..

[51] Avgoustakis K. (2002). PLGA–mPEG nanoparticles of cisplatin: In vitro nanoparticle degradation, in vitro drug release and in vivo drug residence in blood properties. J. Control. Release.

[52] Bigham A., Foroughi F., Motamedi M., Rafienia M. (2018). Multifunctional nanoporous magnetic zinc silicate-ZNFE2O4 core–shell composite for bone tissue engineering applications. Ceram. Int..

[53] Hildebrandt B. (2002). The cellular and molecular basis of hyperthermia. Crit. Rev. Oncol..

[54] Goldstein L.S., Dewhirst M.W., Repacholi M., Kheifets L. (2003). Summary, conclusions and recommendations: Adverse temperature levels in the human body. Int. J. Hyperth..

[55] Dokoumetzidis A., Macheras P. (2006). A century of dissolution research: From Noyes and Whitney to the Biopharmaceutics Classification System. Int. J. Pharm..

